# Cytokine Response Associated with Hepatitis C Virus Clearance in HIV Coinfected Patients Initiating Peg Interferon-α Based Therapy

**DOI:** 10.4084/MJHID.2016.003

**Published:** 2016-01-01

**Authors:** Truong Tam Nguyen, Reihani Niloofar, Pierre-Alain Rubbo, Kuster Nils, Karine Bollore, Jacques Ducos, Georges-Philippe Pageaux, Jacques Reynes, Philippe Van de Perre, Edouard Tuaillon

**Affiliations:** 1 Université Montpellier 1, INSERM U 1058, 34394 Montpellier, France.; 2 Pham Ngoc Thach University of Medicine, Thanh Thai, Ho Chi Minh City, Vietnam.; 3 CHU Montpellier, Département d’Hépato-Gastro-Entérologie, 34295 Montpellier, France.; 4 CHU Montpellier, Département des Maladies Infectieuses et Tropicales, 34295, France.; 5 CHU Montpellier, Département de Bactériologie - Virologie, 34295 Montpellier, France.

## Abstract

**Background:**

Treatment of hepatitis C virus (HCV) infection based on peginterferon-α (pegIFNα) and ribavirin induces important changes in cytokine release and T cell activation.

**Objective:**

Immune response to pegIFNα-ribavirin therapy was explored in patients coinfected by HCV and HIV.

**Methods:**

Concentrations of 25 cytokines and CD8^+^ T cell activation were monitored in HCV/HIV coinfected patients classified as sustained virological responders (SVR, n=19) and non-responders (NR, n=11).

**Results:**

High pretreatment concentrations of IP-10 (CXCL-10) and MCP-1 (CCL-2) were associated with a poor anti-HCV response. PegIFNα-ribavirin therapy increased CD8^+^ T cell activation and induced significant changes in levels of eleven cytokines related to both Th1 and Th2 responses in SVR (IL-1β, IL-1RA, IL-4, IL-5, IL-6, IL-7, IL-12p40/70, IL-13, IP-10, eotaxin, MCP-1) but of only six cytokines in NR (IL-1β, IL-2, IL-5, IL-12p40/70, IL-13, eotaxin). The highest rise in MIP-1β and MCP-1 levels was observed four weeks after anti-HCV treatment initiation in SVR compared to NR (p=0.002 and p=0.03, respectively), whereas a decrease in IL-8 concentration was associated with treatment failure (p= 0.052).

**Conclusions:**

Higher and broader cytokine responses to pegIFNα-ribavirin therapy were observed in SVR patients compared to NR. Changes in IL-8, MIP-1β, and MCP-1 serum concentrations may be associated with efficacy of pegIFNα- and ribavirin-based therapies in patients coinfected by HCV and HIV.

## Introduction

Infection by hepatitis C virus (HCV) and HIV shared common routes of transmission and thus confections with the two viruses are frequent. HCV infection is seen in 15–30% of HIV-infected patients in Western Europe, with almost 20 000 HIV-infected individuals chronically infected with HCV in France.^1^,^2^ Since the introduction of antiretroviral therapy (ART), HCV-related liver diseases have become leading causes of morbidity and mortality in HIV-infected individuals.^3^,^4^

Faster progression to cirrhosis has been observed in patients coinfected with HCV and HIV by comparison with subjects infected with HCV alone.^5^ HIV/HCV coinfection is also associated with higher HCV viral levels in serum.^6^ Finally, ART-related adverse events are more frequent in HCV/HIV coinfected individuals, with increased difficulties in optimally treating HIV infection.^7^,^8^

Until recently, a combination of pegIFN and ribavirin has been the standard-of-care for treatment of chronic HCV patients. In coinfected individuals -ribavirin therapy is less efficacious than in single HCV infection.^9^,^10^ Approximately 30% of patients receiving a combination of ribavirin with pegIFN have a sustained virological response when infected with HCV genotype 1 and 4.^9^ This rate increases to 60% when genotype 2 and 3 are involved.^10^ Most of the current anti-HCV therapeutic options remained based on pegIFN and/or ribavirin in addition to protease inhibitors or NS5A or NS5B inhibitors.^11^

PegIFN constitutes an active immunotherapy triggering innate immune response and T cell activation. The cytokine response to pegIFNα-based regimens plays probably an important role in treatment outcome. IFNα stimulates Th1 cells and limits secretion of Th2 related cytokines such as IL-4 or IL-10.^12^ IFNα signals through Janus kinase signal transducer, an activator of transcription pathway, and induction of IFN-stimulated genes.^13^ Ribavirin synergies this effect by reducing IL-10 production, enhancing autocrine IFN-β and IL-8 secretion.^14^–^16^ Several immune factors are predictive of virological response on pegIFNα-ribavirin-based therapy: pretreatment concentration of IFNα-inducible protein 10 (IP-10, also called CXCL-10), polymorphisms near the interleukin-28B gene coding for the IFNα-3, and CD4^+^ T cell count in HIV/HCV coinfected individuals.^18^,^19^ Progressive impairment of immune functions and T cell exhaustion induced by prolonged exposure to virus antigens are fundamental features of HIV infection. Inhibitory networks, such as the programmed cell death protein-1 (PD-1) and IL-10 play a key role in this process.^20^,^21^ PD-1 is upregulated on CD8^+^ and CD4^+^ T cells and mediates a dysfunction partially reversible on ART.^22^–^24^ PD-1 signalling impacts the expression of both Th1 and Th2 cytokines but limits particularly the capacity of Th1 and IFNγ secretion by CD4^+^ and CD8^+^ T cells.^21^

Although HCV clearance depends on immune response induced by pegIFNα-ribavirin therapy, the dynamics of cytokine response and T cell activation in individuals coinfected with HIV and HCV remain poorly characterized.^25^ In this study, we monitor serum concentration of 25 cytokines alongside with the level of CD8^+^ T cell activation in HIV/HCV coinfected patients initiating anti-HCV therapy.

## Methods

### Patient samples

Patients coinfected by HIV/HCV followed at Montpellier University Hospital and initiating anti-HCV therapy were included consecutively between January 2007 and January 2010 after providing written informed consent. The study was approved by the local institution ethical committee (DC-2011-1405). Chronic hepatitis was proved by the presence of serum HCV antibodies and detectable viral RNA. HCV genotype, HCV and HIV-1 viral loads, CD4^+^ T cell count, and liver enzyme levels were all determined using standard procedures. All patients received pegIFNα2a 180 microg/week plus ribavirin 800 to 1,200 mg daily; patients infected with HCV genotype 2 and 3 received 800 mg daily and genotype 1 and 4 received a weight based dose: 1,000 mg/day for persons less than 75 kg and 1200 mg/day over 75 kg.

Subjects were ranged into two distinct groups based on HCV response to therapy:

- Sustained virological responders (SVR) when serum HCV RNA was undetectable 24 weeks after completing therapy, and
- non responders (NR) when HCV RNA decrease was < 2 log copies/mL at week 12 or when HCV RNA was detectable at the end of treatment.
- Patient relapsing in the 24 weeks period after therapeutic cessation were excluded from the study.

### Routine laboratory testing

Serum HCV RNA was quantified using COBAS AmpliPrep/COBAS TaqMan HCV assay (Roche Diagnostic Systems). HCV genotypes were determined using INNO-LiPA HCV II test (Innogenetics). Alanine aminotransferase (ALT) dosages were executed using standard methods.

### Quantitation of serum cytokine concentrations

Cytokines were quantified in serum samples obtained before initiation of treatment, and four weeks after the start of PegIFNα-based therapy. A multiplexed microbead assay was used according to manufacturer’s instructions (cytokine twenty-five-plex kit, Life Technologies Ltd, Paisley, UK) and an FIDIS apparatus (BMD). Twenty five cytokines were quantified in duplicate: IL-1β, IL-1RA, IL-2, IL-2R, IL-4, IL-5, IL-6, IL-7, IL-8, IL-10, IL-12p40/70, IL-13, IL-15, IL-17, eotaxin, GM-CSF, IFN-α, IFN-γ, IP-10, MCP-1, MIG, MIP-1α, MIP-1β, RANTES, and TNF-α. Data were analyzed using the MLX-Booster program (BMD). Mean concentrations (pg/ml) of cytokines were all superior to the detection limits, defined as the mean background value plus 2 SD.

### Analysis of CD8^+^/CD38^bright^ T cells

Activation of CD8^+^ T cells was assessed by flow cytometry analysis on EDTA-treated fresh whole blood using FC 500 apparatus (Beckman Coulter, Miami, Florida). T cell activation was explored at day 0, week 4 and week 12 after initiation of pegIFNα-ribavirin therapy. The expression of CD38^bright^ on CD8^+^ T cells was analyzed as previously described using a two-colour staining with anti-CD8 and anti-CD38 conjugated to fluorescein isothiocyanate (FITC) and phycoerythrin (PE), respectively (Beckman Coulter).^26^ The positive threshold for CD38^bright^ analysis was established using the CellQuant CD38/CD8 kit for quantitation of CD38 cell surface expression (BioCytex, Marseille, France) and was defined as 8,500 CD38 binding sites/cells. The CD8^+^/CD38^bright^ values were expressed as the percentage of CD38^bright^ cells from the CD8^+^ T cell populations.

### Statistical Analysis

The Mann–Whitney U test and Kruskal–Wallis test were used to analyze continuous variables where appropriate. The Friedman test was used to evaluate changes in serum cytokine levels over time. A mixed model was used to analyze the association between T cell activation during anti-HCV therapy and therapeutic response. Statistical analyses were done using SPSS software version 18.0J.

## Results

### Clinical characteristics at baseline

Thirty HCV/HIV coinfected patients receiving pegIFNα-ribavirin therapy were included in the study. Nineteen (63%) were classified as SVR and eleven (37%) as NR. Patient characteristics are shown in Table 1.

### Pretreatment levels of soluble and cellular inflammatory markers

IP-10 levels were higher in the NR group than in the SVR group, *P* = 0.021 (Table 2). Individuals from the NR group also had higher pretreatment concentrations of MCP-1, *P* = 0.0009. A trend for a higher serum concentration of IL-8 was also observed in patients of the NR group compared to the SVR group, *P* = 0.106.

### Cytokines change after therapeutic initiation of HCV treatment

**Figure 1:**
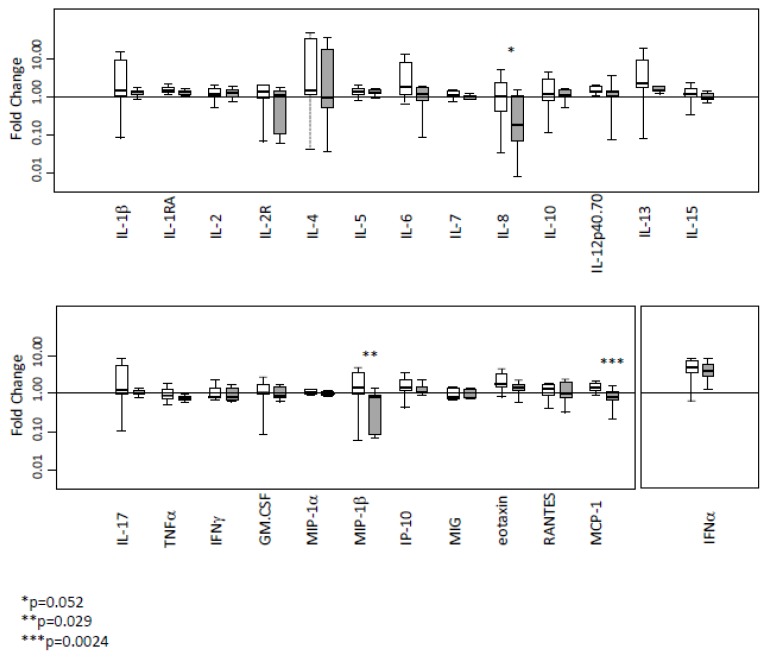
Impact of anti-HCV therapy on cytokine concentrations in serum of NR and SVR. Fold-changes in cytokine levels following pegIFNα-ribavirin therapy initiation in SVR (white bars) and NR (gray bars). Results are expressed as fold changes in cytokine concentrations between week 0 (pretreatment) and week 4 (four weeks of pegIFNα-ribavirin therapy). ^*^p=0.052, ^**^p=0.029, ^***^p=0.0024.

Initiation of anti-HCV treatment elicited IFNα rise in both SVR and NR patients (Figure 1 **and Supplemental Table 1**). This enhancement of IFNα in serum was similar in the two groups. Besides IFNα, the anti-HCV therapy induced a significant increase in the serum levels of eleven cytokines in SVR patients, including cytokines related to Th1-dominant immune responses (IL-12p40/70, IP-10), Th2-type cytokines (IL-4, IL-5, and IL-13), and pro-inflammatory cytokines (IL-1β, IL-1RA, IL-6, IL-7). By comparison, levels of only six cytokines rose in the NR group (**Supplemental Table 1**). Finally, when results were analyzed without consideration of the HCV therapeutic response (combining SVR and NR patients), we also observed an increase of IL-2, IL-10, and RANTES concentrations four weeks after initiation of anti-HCV treatment (**Additional file 1**).

### Comparison of the cytokine level changes induced by pegIFNα-ribavirin between SVR and NR group

Changes in cytokines were compared among SVR and NR using the ratio between baseline and four weeks of treatment (Figure 1). A sharp increase of MCP-1 and MIP-1β concentration was observed after initiation of anti-HCV therapy in the SVR group whereas the value decreased or remained stable in the NR group (p=0.0024 and p=0.029, respectively). Furthermore, the IL-8 concentration remains stable after a one-month period of pegIFNα-ribavirin therapy in most of the patients responding to anti-HCV treatment, whereas this concentration declined in NR patients (p=0.052).

### Changes in T cell activation over the three-months period of therapy initiation

**Figure 2:**
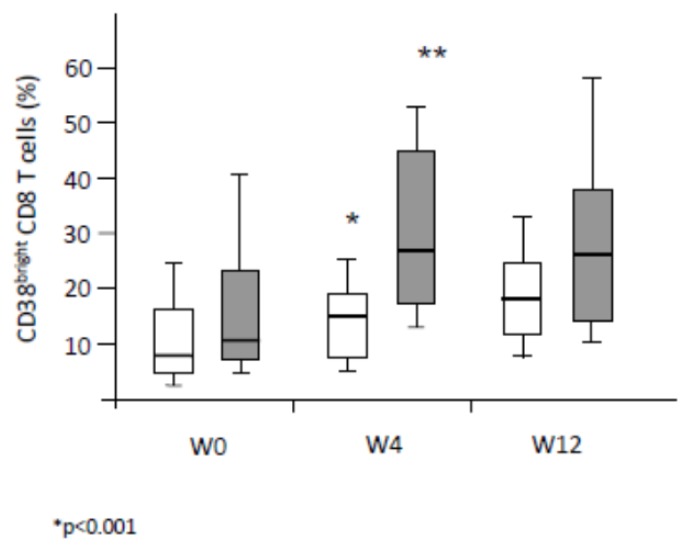
Changes in T cell activation over a 3-month period of pegIFNα-ribavirin therapy. Fold-changes in CD38^bright^ expression on CD8 T cells following pegIFNα-ribavirin therapy initiation in SVR (white bars) and NR (gray bars). A significant increase of CD38^bright^ expression between two consecutive time periods is indicated: ^*^p<0.001.

The impact of pegIFNα-ribavirin on T cell activation was explored at day 0, week 4 and week 12 (Figure 2). CD8^+^ T cell activation before the treatment tends to be higher in NR patients compared to the SVR patients, (p=0.066, Figure 2). Anti-HCV therapy induced a major increase in CD38^bright^ expression on CD8 T^+^ cells in the two groups of patients (p<0.001). No significant differences were observed between the two groups in the slope of CD38 cell surface expression of CD8^+^ T cells suggesting that the over time changes of this activation marker not be associated with a better immune response to therapy.

## Discussion

Although the virological response to pegIFNα/ribavirin-based therapy is intimately associated with the host immunity, the monitoring of HCV treatment is based on the serum HCV-RNA level decay regardless of the immune response. In this study, CD8^+^ T cell activation and levels of 25 cytokines were analyzed in HIV/HCV coinfected patients with favourable versus unfavourable therapeutic outcome.

PegIFNα/ribavirin administration induced a dramatic change in serum cytokine concentrations and CD8^+^ T-cell activation level. We observed that the HCV clearance under pegIFNα/ribavirin therapy was associated with broader and higher cytokine responses in SVR than in NR.

Multianalyte assays make possible to analyze a wide an array of cytokines using a small sample volume rapidly. In the present study, the level of 25 cytokines and CD8^+^ T-cell activation were analyzed at baseline and week four after the initiation of pegIFNα/ribavirin therapy. Comparison between different multiplex bead assays has shown variable agreement among kits evidencing that absolute cytokine concentrations differ across commercial assays.^27^,^28^ As a consequence, multisite comparisons between cytokine concentrations obtained with different methods are difficult to interpret because of the lack of standardization. However, these methods are useful for longitudinal studies, and a similar rank order of cytokine concentrations between samples is preserved between the different commercial kits. Hence, we considered comparisons based on fold change under pegIFNα-ribavirin therapy as a valuable method for identifying a predictor of therapeutic success or failure. Even if interferon-free regimen progressively substitute pegIFNα-containing regimen for treatment of hepatitis C it remains of particular importance to describe immune response after initiation of pegIFNα. Indeed, IFNα remains the only drug approved for treatment of chronic hepatitis D and the only treatment used for a limited period for hepatitis B.

Baseline serum concentration of IP-10 and MCP-1 (monocyte chemotactic protein 1) were higher in NR than in SVR. A significant increase was observed after treatment initiation in 15 out of 25 soluble biomarkers tested. The strongest increased was observed for IFNα as an expectable consequence of the anti-HCV therapy, with more than 4-fold rise between week 0 and week 4. Increase in cytokine concentrations after pegIFNα-ribavirin administration was observed for pro-inflammatory- and Th1-related cytokines such as IL-12 or IP-10, but also for Th2-related cytokines such as IL-4 or IL-13. Activation of T cells accompanied this phenomenon as shown by the increased cell surface expression of CD38 on CD8^+^ T cells.

Based on therapeutic outcome distinct cytokine patterns were identified. Differences in cytokine concentrations were observed at baseline and following anti-HCV initiation. In agreement with previous studies in individuals coinfected we observed the pretreatment serum level of IP-10 was higher in the NR group than in the SVR group.^19^ Circulating IP-10 concentration is well correlated with intrahepatic IP-10 messenger RNA expression during chronic HCV infection.^29^ The high baseline hepatic IFN-stimulated genes expression is associated with a lower chance to be SVR to IFN-based therapy when HIV-uninfected.^30^–^33^ Interestingly, the pre-treatment MCP-1 concentration was also found at a higher concentration in the NR group. This pro-inflammatory chemokine is involved in the migration and infiltration of monocytes and CD4^+^ memory T lymphocytes. Intra-hepatic and serum MCP-1 levels are associated with liver inflammation and fibrosis during chronic HCV infection.^34^,^35^ MCP-1 plays a significant role in the recruitment of monocytes by interacting with their cell-surface adhesion-molecules that are over expressed during chronic HCV infection.^36^ Association between MCP-1 baseline level, and response to pegIFNα-ribavirin therapy has been recently reported in HIV-uninfected patients.^37^ Both chronic HCV and HIV infections induce expression and release of MCP-1 in humans.^38^,^39^ HIV proteins like gp120^40^ and transactivator protein Tat^41^,^42^ have shown strong immunomodulatory capacity through MCP-1 stimulation. MCP-1 has also been shown to promote Th2 responses by polarizing Th0 cells towards a Th2 phenotype^43^ and is thought to be one of the key factors involved in HIV pathogenesis.^44^ Hence, MCP-1 pretreatment level may be associated with the promotion of the Th1 dominant response and HCV clearance under pegIFNα-ribavirin therapy in HIV/HCV coinfected patients.

Cytokine response to pegIFNα/ribavirin therapy differed qualitatively between SVR and NR. The cytokine response appeared more restricted in NR than in SVR since only six versus 11 cytokines rise significantly following the commencement of anti-HCV therapy. As a possible consequence of high IP-10 and MCP-1 baseline level, the boost of circulating IP-10 and MCP-1 levels was not observed in the NR after four weeks of pegIFNα-ribavirin treatment. Circulating IP-10 is considered as a marker of the pre-therapeutic activation level of IFN-stimulated genes. The defect in IP-10 response observed four weeks after starting pegIFNα-ribavirin treatment can be viewed as a marker of the overexpression of IFN-stimulated genes leading to a poor capacity response to exogenous IFN injections.

**Figure:**
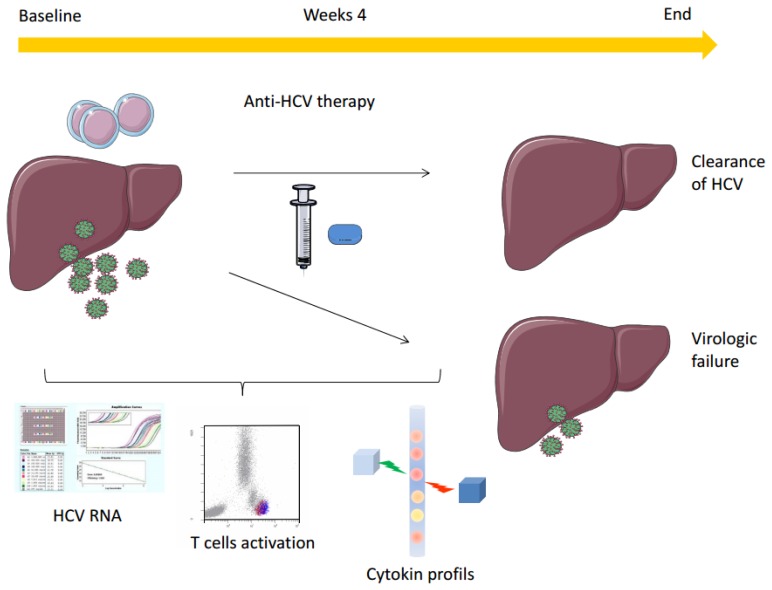


Four weeks after initiation of pegIFNα-ribavirin, a higher MIP-1β (macrophage inflammatory protein-1β) and MCP-1 secretions were observed in SVR when compared to NR. Of interest, a trend for a paradoxical decrease of MIP-1β was also observed at the same time in the NR group. The concentration of MIP-1β was halved in the NR group at week four compared to baseline whereas MCP-1β level tends to increase in the SVR group. This observation is consistent with a previous study showing that HCV clearance is more frequent in patients experiencing strong MIP-1α and MIP-1β response to pegIFNα-based therapy.^45^,^46^ MIP-1β (also named CCL4) is a chemokine linked to the Th1 response. MIP-1β is involved in T cell recruitment mediated by CCR5 and CCR1 in the liver of HCV infected patients.^38^ The amplitude of MCP-1 variation in response to pegIFNα/RBV is probably dependent on baseline MCP-1 level as for IP-10. IL-8 dynamic response to pegIFNα-ribavirin therapy also appears different in the two groups. IL-8 serum levels tend to be elevated at baseline in NR and to diminish following pegIFNα-ribavirin initiation. High IL-8 mRNA is involved in liver inflammation^47^–^49^ and inhibition of the IFNα antiviral response *in vitro*.^50^ Pretreatment level of IL-8 has also been found associated with poor virological response to pegIFNα-ribavirin therapy.^51^ Evolution of IL-8 concentration may reflect the combined effect of pegIFN plus ribavirin since ribavirin induces its secretion through the activation of activator protein 1.^14^

In this study, relapse patients were not included in the group of patients that did not recover from HCV under pegIFNα-ribavirin. Subjects who experience relapse had patterns of virological response different from NR with a rapid decline in HCV RNA during the first week of treatment.^52,53^ Hence, relapse patients should be explored a part from NR and SNR groups.

Our study had limitations related to its retrospective nature and the small number of subjects included. HCV genotypes and fibrosis stage were not taken into account in the analysis and IL-28 polymorphism, which is also known to predict the hepatic responsiveness to IFN-based therapy, was not assessed. The study was not designed to establish clinical performances of immunological markers useful for HCV therapeutic prediction and/or monitoring but showed that different ongoing trends of cytokine response to pegIFNα/ribavirin can be observed in SVR and NR patients infected with HIV.

## Conclusions

HIV/HCV coinfected patients, showing a high IP-10 and MCP-1 baseline level alongside with a reduced capacity to produce or to maintain broader secretion of cytokines, including MIP-1β, IL-4, IL-6 or IL-8, may have a poor response to regimens based on Peg-IFN and ribavirin. Anti-HCV treatment based on drugs having immunomodulating activities may benefits from immunomonitoring using multiplex techniques.

## 

## Figures and Tables

**Table 1:** Patient characteristics

	All (n=30)	SVR (n=19)	NR (n=11)*
**Age**	45	44	47
**CD4^+^** **T cells (/μl)**	350 (50–1076)	378(128–1076)	328 (50–715)
**Nadir CD4 T cells (/μl)**	122.5 (32–598)	124 (33–598)	119.5 (32–324)
**ALAT (IU/L)**	48.5 (13–172)	40.5 (13–172)	56.5 (24–133)
**VL HCV S0 (Log_10_** **IU/ml)**	5.96 (5.43–6.68)	5.49 (4.12–5.81)	6.68 (5.98–6.84)
**VL HIV S0 (IU/l)**	0 (0–290)	0 (0–290)	0 (0–118)
**HCV Genotypes 1/2/3/4**	21/2/7/1	10/1/8/0	10/0/0/1
**Liver fibrosis (F≥2)**	22	13	9

* Seven subjects had a reduction of less than 2 log_10_ in HCV RNA after 12 weeks of therapy (null responders), and four subjects had a reduction of more than 2 log_10_ (partial responders).

**Table 2:** Pre-treatment Cytokine concentrations in serum from SVR and NR patients

Cytokines	SVR pg/ml mean [CV]	NR pg/ml mean [CV]	p-value (Wilcoxon)
**IL-1β**	12.9 [ 34.2 ]	10.84 [ 4.9 ]	0.093
**IL-1RA**	1556.0 [ 198.0 ]	1506.0 [ 123.0 ]	0.779
**IL-2**	6.2 [ 2.4 ]	5.8 [ 2.6 ]	0.425
**IL-2R**	837.0 [ 812.2 ]	884.1 [ 155 ]	0.101
**IL-4**	8.0 [ 177.9 ]	8.0 [ 173.5 ]	0.338
**IL-5**	2.3 [ 0.5 ]	2.3 [ 0 ]	0.616
**IL-6**	4.6 [ 2.2 ]	5.0 [ 23.0 ]	0.353
**IL-7**	18.9 [ 2.3 ]	20.8 [ 4.3 ]	0.142
**IL-8**	22.5 [ 73.8 ]	128.1 [ 707.7 ]	0.106
**IL-10**	3.6 [ 1.1 ]	3.1 [ 2.0 ]	0.777
**IL-12p40/70**	321.5 [ 55.8 ]	318.3 [ 56.5 ]	0.948
**IL-13**	11.0 [ 4.1 ]	11.0 [ 2.7 ]	0.702
**IL-15**	33.2 [ 9.4 ]	33.2 [ 3.1 ]	0.863
**IL-17**	24.8 [ 9.3 ]	24.0 [ 8 ]	0.73
**TNFα**	8.8 [ 2.6 ]	8.8 [ 1.1 ]	0.762
**IFNγ**	5.2 [ 1.3 ]	6.1 [ 0.8 ]	0.621
**GM.CSF**	18.4 [ 9.2 ]	15.3 [ 13.8 ]	0.482
**MIP-1α**	119.4 [ 1.8 ]	120.6 [ 6.8 ]	0.262
**MIP-1β**	150.4 [ 24.3 ]	150.4 [ 23.6 ]	0.88
**IP-10**	64.3 [ 32.9 ]	132.0 [ 58.8 ]	**0.021**
**MIG**	458.6 [ 5.9 ]	458.6 [ 4.0 ]	0.497
**Eotaxin**	75.5 [ 30.7 ]	72.9 [ 29.4 ]	0.813
**RANTES**	10540.0 [ 6413.0 ]	9350.0 [ 10922.0 ]	0.683
**MCP-1**	347.9 [ 91.2 ]	442.6 [ 145.7 ]	**0.009**
**IFNα**	143.0 [ 21.3 ]	143.0 [ 1.4 ]	0.477
