# Supplementary material for: Cytokine Response Associated with Hepatitis C Virus Clearance in HIV Coinfected Patients Initiating Peg Interferon-α Based Therapy
Source: Mediterr J Hematol Infect Dis. 2016 Jan 1;8(1):e2016003. doi: 10.4084/MJHID.2016.003 (PMC4696469; doi:10.4084/MJHID.2016.003)
Supplement: Supplementary file 1 [file Supplemental_table_1_Tuaillon.pdf]

Supplemental table 1: Fold changes in serum cytokines level four week after initiation of pegIFNα-ribavirin therapy.

**Total (n=30)**			**SVR (n=19)**		**NR (n=11)**	
Cytokine	Median [95%IC]	p-value	Median [95%IC]	p-value	Median [95%IC]	p-value
IL1beta	2.05 [1.21 - 4.86]	**<0.001**	4.42 [1.15 - 6.17]	**0.0053**	1.32 [1.11 - 4.09]	**0.0068**
IL1RA	1.38 [1.3 - 1.54]	**<0.001**	1.43 [1.32 - 1.63]	**<0.001**	1.35 [0.99 - 1.57]	0.0537
IL2	1.3 [1.07 - 1.54]	**0.0066**	1.3 [1 - 1.65]	0.0546	1.29 [1 - 6.1]	**0.042**
IL2R	1.26 [0.79 - 6.03]	0.2621	1.38 [0.83 - 8.44]	0.0955	0.93 [0.42 - 7.78]	0.8311
IL4	16.73 [1.19 - 19.42]	**0.0104**	16.81 [1.28 - 19.67]	**0.0058**	1.21 [0.49 - 21.1]	0.7596
IL5	1.34 [1.23 - 1.47]	**<0.001**	1.31 [1.16 - 1.52]	**0.001**	1.37 [1.18 - 1.77]	**0.0067**
IL6	1.65 [1.23 - 7.08]	**0.0013**	2.11 [1.33 - 10.58]	**0.001**	1.23 [0.69 - 6.36]	0.5195
IL7	1.12 [1.02 - 1.5]	**0.0237**	1.14 [1.03 - 1.63]	**0.0124**	1.03 [0.89 - 4.1]	0.6247
IL8	0.95 [0.56 - 2.39]	0.9032	1.26 [0.68 - 3.13]	0.49	0.58 [0.09 - 2.9]	0.1748
IL10	1.43 [1.02 - 2.35]	**0.0417**	1.67 [0.92 - 3.15]	0.0948	1.21 [0.89 - 3.45]	0.2061
IL12p40.70	1.44 [1.31 - 1.61]	**<0.001**	1.48 [1.29 - 1.8]	**<0.001**	1.42 [1.22 - 1.66]	**0.001**
IL13	1.99 [1.61 - 8.89]	**<0.001**	2.24 [1.63 - 9.6]	**<0.001**	1.66 [1.38 - 12.04]	**0.0038**
IL15	1.13 [0.95 - 1.34]	0.1812	1.19 [0.97 - 1.49]	0.08	0.97 [0.81 - 4.72]	0.8984
IL17	1.22 [0.99 - 4.76]	**0.0799**	1.9 [0.99 - 5.18]	0.0663	1.04 [0.76 - 4.74]	0.8385
TNFa	0.86 [0.76 - 1.12]	0.2286	0.94 [0.78 - 1.21]	0.8596	0.77 [0.68 - 14.39]	0.0674
IFNg	1.04 [0.78 - 1.21]	0.5784	1.03 [0.78 - 1.22]	0.732	1.06 [0.73 - 1.41]	0.6889
GM.CSF	1.24 [0.96 - 1.57]	**0.0712**	1.34 [0.89 - 2]	0.1025	1.16 [0.8 - 1.44]	0.624
MIP-1a	1.06 [0.74 - 1.16]	0.7096	1.08 [0.73 - 1.24]	0.6026	0.99 [0.64 - 1.16]	0.9188
MIP-1b	1.08 [0.71 - 2.54]	0.7922	1.37 [0.84 - 5.52]	0.1232	0.56 [0.1 - 1.09]	0.083
IP10	1.46 [1.18 - 1.94]	**6,00E-04**	1.6 [1.19 - 2.56]	**0.0033**	1.24 [0.91 - 1.96]	0.1016
MIG	1.07 [0.77 - 1.09]	0.3762	1.04 [0.76 - 1.1]	0.5062	1.08 [0.73 - 1.24]	0.6247
EOTAXIN	1.95 [1.53 - 2.45]	**<0.001**	2.24 [1.57 - 2.98]	**<0.001**	1.55 [1.16 - 2.23]	**0.0068**
RANTES	1.33 [1.01 - 1.89]	**0.0427**	1.31 [0.97 - 3.19]	0.0874	1.4 [0.73 - 2.04]	0.2783
MCP1	1.23 [1.04 - 1.47]	**0.0145**	1.42 [1.2 - 1.97]	**<0.001**	0.87 [0.69 - 1.24]	0.5771
IFNa	4.89 [3.85 - 6.12]	**<0.001**	5.06 [3.83 - 6.71]	**<0.001**	4.6 [2.95 - 8.51]	**0.001**
